# Unveiling metabolic pathways in the hyperglycemic bone: bioenergetic and proteomic analysis of the bone tissue exposed to acute and chronic high glucose

**DOI:** 10.1186/s10020-025-01251-0

**Published:** 2025-05-17

**Authors:** Rita Araújo, Raquel L. Bernardino, Mariana P. Monteiro, Pedro S. Gomes

**Affiliations:** 1 BoneLab, Faculdade de Medicina Dentária, Universidade do Porto, Rua Dr. Manuel Pereira da Silva, 4200-393 Porto, Portugal; 2 LAQV/REQUIMTE, Faculdade de Medicina Dentária, Universidade do Porto, Rua Dr. Manuel Pereira da Silva, 4200-393 Porto, Portugal; 3 Endocrine and Metabolic Research, UMIB-Unit for Multidisciplinary Research in Biomedicine, ICBAS-School of Medicine and Biomedical Sciences, University of Porto, Porto, Portugal; 4 Laboratory for Integrative and Translational Research in Population Health (ITR), University of Porto, Porto, Portugal

**Keywords:** Diabetic bone disease, High glucose, Bioenergetics, Protein expression, Ex vivo model, Seahorse analyzer, Mass spectrometry

## Abstract

**Background:**

Bone fragility due to poor glycemic control is a recognized complication of diabetes, but the mechanisms underlying diabetic bone disease remain poorly understood. Despite the importance of bioenergetics in tissue functionality, the impact of hyperglycemia on bone bioenergetics has not been previously investigated.

**Objective:**

To determine the effects of high glucose exposure on energy metabolism and structural integrity in bone tissue using an ex vivo organotypic culture model of embryonic chick femur.

**Methods:**

Femora from eleven-day-old Gallus gallus embryos were cultured for eleven days under physiological glucose conditions (5.5 mM, NG), chronic high glucose exposure (25 mM, HG-C), or acute high glucose exposure (25 mM, HG-A). Bioenergetic assessments (Seahorse assays), proteomic analysis (liquid chromatography-mass spectrometry), histomorphometric and microtomographic evaluations, and oxidative stress measurements (carbonyl content assay) were performed. Statistical analyses were conducted using IBM® SPSS® Statistics (v26.0). The Mann–Whitney nonparametric test was used for group comparisons in microtomographic analysis, ALP activity, and carbonyl content assays. For Seahorse assay results, ANOVA with Tukey's post-hoc test was applied after confirming data homoscedasticity with Levene’s test.

**Results:**

Chronic high glucose exposure reduced bone mineral deposition, altered histomorphometric indices, and suppressed key osteochondral development regulators. Acute high glucose exposure enhanced glycolysis and oxidative phosphorylation, while chronic exposure caused oxygen consumption uncoupling, increased ROS generation, and downregulated mitochondrial proteins critical for bioenergetics. Elevated oxidative stress was confirmed in the chronic high glucose group.

**Conclusion:**

Chronic high glucose exposure disrupted bone bioenergetics, induced mitochondrial dysfunction, and compromised bone structural integrity, emphasizing the metabolic impact of hyperglycemia in diabetic bone disease.

## Background

Diabetes mellitus (DM) is a metabolic disease characterized by chronic hyperglycemia, leading to micro and macrovascular injury that results in multiple end-organ damage and disease complications, significantly reducing patients’ quality of life and imposing a substantial socio-economic burden (Skyler 2004; Schmidt 2018). DM is a heterogeneous group of diseases classified into different clinical categories with distinct etiologies that can involve a complex interplay of genetic, autoimmune, and environmental factors, leading to widespread metabolic dysfunction and pathological alterations in various tissues and organs, including bone tissue (Skyler 2004; Schmidt 2018; Committee ADAPP. 2,2023). Diabetic bone disease, irrespective of the type of diabetes, is associated with significant metabolic and structural changes. These changes result in increased skeletal fragility, a higher risk of fracture, delayed healing, and impaired bone quality, collectively compromising the structural integrity and functionality of bone (Stefano et al. 2016). Despite extensive research, the precise mechanistic drivers underlying these alterations remain largely unknown (Schmidt 2018).

A key area of interest in understanding these alterations is cellular bioenergetics, which encompasses the metabolic processes responsible for supplying cellular energy (Stefano et al. 2016). These processes are essential for maintaining cellular and tissue functionality and primarily rely on glycolysis and oxidative phosphorylation (OXPHOS) (Ferreira et al. 2003; Hartman et al. 2014; Wefers Bettink et al. 2019). Glycolysis, occurring in the cytosol under both anaerobic and aerobic conditions, converts glucose into pyruvate, yielding adenosine triphosphate (ATP) and providing essential intermediates for anabolic processes (Spinelli and Haigis 2018). OXPHOS, occurring within the mitochondria, produces ATP through an electrochemical gradient generated by the electron transport chain, which is dependent on electron donors from the oxidation of acetyl-CoA in the tricarboxylic acid (TCA) cycle (Spinelli and Haigis 2018; Nishikawa et al. 2000). Bioenergetics is, however, tissue-specific and relies on the kinetic properties of the involved enzymes, local tissue factors, and specific energy demands, all of which collectively determine the efficiency and capacity of metabolic pathways (Spinelli and Haigis 2018). While most tissues preferably use OXPHOS for their energetic demands (Esen and Long 2014), in bone, osteoblasts seem to utilize glucose during differentiation via both glycolysis and OXPHOS, with a marked preference for the former (Esen and Long 2014; Wei et al. 2015).

In the context of DM, the bioenergetic profile of bone tissue remains poorly understood. Chronic hyperglycemia impacts cellular bioenergetics in various tissues by disrupting normal metabolic processes and reducing the efficiency of energy metabolism (Chowdhury et al. 2013). Excess glucose leads to the overproduction of reactive oxygen species (ROS) in mitochondria, causing oxidative stress and damaging cellular components (Giri et al. 2018). This oxidative stress impairs mitochondrial function, reducing ATP production efficiency (Giri et al. 2018). Additionally, chronic hyperglycemia may alter metabolic pathways, including glycolysis and the TCA cycle, further hindering energy production (Giri et al. 2018). These disruptions in cellular energy homeostasis can lead to tissue dysfunction and contribute to the development of complications in diabetes, as established for different tissues such as the retina and kidney (Alka et al. 2023; Audzeyenka et al. 2021). Despite these well-documented impacts in different tissues, the bioenergetic profile of bone tissue under hyperglycemic conditions remains largely unknown.

Herein, using an established translational ex vivo model of bone in high glucose conditions, that displays structural and molecular alteration coherent with the ones observed in diabetic patients (Araújo et al. 2022), this study will investigate the effects of hyperglycemic conditions on the bioenergetics of bone tissue by exposing embryonic chick femurs to high glucose levels. Both acute and chronic exposures to hyperglycemia will be conducted, allowing to elucidate the effects associated with the long-term exposure, characteristic of the DM condition. The objectives include assessing mitochondrial function, evaluating glycolysis, and characterizing the tissue proteome to provide comprehensive insights into the potential metabolic dysfunctions induced by hyperglycemia in bone tissue. This approach aims to uncover novel therapeutic targets for bone loss of functionality and enhance the understanding of its underlying bioenergetic mechanisms. By integrating these analyses, we seek to elucidate the specific local alterations in energy metabolism that contribute to the compromised bone quality observed in chronic hyperglycemic conditions.

## Materials and methods

### Ex vivo organotypic culture

The organotypic culture in high glucose condition was established as previously described (Araújo et al. 2022). Briefly, embryonic femora were isolated from eleven-day chicken embryos obtained from a certified local vendor. The embryos were incubated at 37.4ºC and 60% relative humidity in a conventional rotating incubator. After dissection, embryonic femurs were placed in Netwell (Costar®) inserts (440 µm mesh size) in six-well plates and cultured in a standard incubator under a humidified atmosphere of 5% CO_2_ at 37 ºC. Two experimental groups were established: 1) normoglycemic group (NG): femora were cultured in a buffered culture medium—alpha minimum essential medium (α-MEM) supplemented with ascorbic acid (50 µg/mL), amphotericin B (2.5 µg/mL), streptomycin (100 µg/mL), and penicillin (100 units/mL), all purchased from Gibco®—the medium contained 5.5 mM glucose; 2) chronic hyperglycemic group (HG-C): femora were cultured with the described medium for NH conditions, further supplemented with a glucose solution to obtain a final concentration of 25 mM glucose. This glucose concentration was selected based on previous literature, where it has been widely used to model chronic hyperglycemia and its effects on bone tissue (Li et al. 2020). Medium was changed daily for eleven days, until the end of the experimental period, identified in previous studies as the optimal timeframe for observing tissue responses in organotypic cultures (Smith et al. 2014). Upon culture period, femur samples were processed and analyzed accordingly. For selected assays, an acute hyperglycemic condition (HG-A) was established, with femora cultured under NG conditions, and acutely exposed to 25 mM glucose during bioenergetic assays. This group (HG-A) was included to elucidate the differential impacts of acute versus chronic hyperglycemia on the bioenergetic profile. This approach allowed for distinguishing between the immediate effects of high glucose levels and the longer-term adaptations that occur under sustained hyperglycemic conditions.

### Microtomographic analysis

Microtomographic analysis was conducted on femora upon scanning in the Skyscan 1276 System (Bruker®, Belgium) at 40 kv and 200 µA. The obtained three-dimensional datasets were reconstructed with NRecon 1.7.4.2 (Bruker®, Belgium) software. The following histomorphometric indexes were determined, using CTanalyser 1.19.31 (Bruker®, Belgium) software: Total Volume (mm^3^), Bone Volume (mm^3^), Bone Volume Fraction (BV/TV) (%). The whole femur was used as volume of interest (VOI). Representative images were generated with CTvox 2.3.2.1 software (Bruker®, Belgium). The analysis was conducted in five samples from each group.

### Alkaline phosphatase activity

Alkaline phosphatase (ALP) activity was quantified in whole bone lysates upon tissue processing in 0.1% Triton X-100 for 5 min. The hydrolysis process of p-nitrophenylphosphate in alkaline buffer solution at pH of 10.3 and a temperature of 37 ⁰C was determined, through colorimetric evaluation of the biproduct (p-nitrophenol) in an ELISA plate reader at 400 nm (Synergy HT, Biotek). Results were normalized to total protein content. Total protein quantification was performed using the Lowry method, with the RC/DC Biorad® kit, according to the manufacturer´s instructions. This assay was performed in triplicates.

### Seahorse assay

The oxygen consumption rate (OCR) was determined in the grown femurs incubated with Seahorse assay medium (DMEM medium, pH = 7.4, Agilent Technologies, Santa Clara, CA, USA) supplemented with 1 mM pyruvate, 2 mM glutamine, and 5.5 mM or 25 mM glucose, for 45 min, at 37 °C in a CO_2_-free incubator, and recorded in real time, using a Seahorse XF24 analyzer (Agilent Technologies, Santa Clara, CA, USA). In addition to the NG and HG-C groups, an acute exposure to high glucose (HG-A) was established upon the injection of 25 mM glucose into the wells harboring femora cultured in normoglycemic conditions. Sham injection was performed for groups NG and HG-C. During the assay, mitochondrial function was modulated by Seahorse XF Cell Mito Stress Test Kit. There are performed sequential injections of the following compounds: 15 µM oligomycin, 8 µM carbonyl cyanide-4 (trifluoromethoxy) phenylhydrazone (FCCP), and a mix of antimycin A (2 µM) and rotenone (4 µM). During the assay, the culture medium was supplemented with 5.5 mM glucose for the NG and HG-A groups and 25 mM glucose for the HG-C group, to match organotypic culture conditions.

The Glycolytic Stress Assay kit was conducted to determine the extracellular acidification rate (ECAR) associated with femora glycolytic activity. Bone tissues were incubated with Seahorse assay medium (DMEM medium, pH = 7.4, Agilent Technologies,Santa Clara, CA, USA) supplemented with 1 mM pyruvate and 2 mM glutamine, for 45 min at 37 °C in a CO2-free incubator. Glycolytic function was modulated by sequential injection of glucose at concentrations of 5.5 mM for the NG group and 25 mM for the HG-A and HG-C groups, followed by oligomycin (15 µM) and 2-Deoxi-D-glucose (2DG) at 100 mM, which inhibits the glycolytic process by targeting the hexokinase enzyme.

Bioenergetic parameters were calculated using Seahorse Analytics platform. Calculations were normalized in relation to total protein content of each sample, which was determined using the DC/ RC protein assay kit™ (Bio Rad), according to manufacturer´s instructions. Six replicates from each group were used for this assay.

### Carbonyl content assay

Protein carbonyl derivatives content was assayed as previously described. Immunodetection of carbonyls was performed using an anti-dinitrophenyl antibody (1:2000; MAB2223, Merck Millipore). The bands were visualized with enhanced chemiluminescence (ECL; Advansta, GRISP), according to the supplier's instructions, and images were recorded using a Molecular Imager Gel Doc XR + System (Bio-Rad) and analyzed with ImageLab (version 5.0, Bio-Rad) (Westermann 2010). The assay was performed in triplicates.

### Proteome characterization

Proteome characterization was performed for femora mid-diaphysis from NG and HG-C groups upon culture period. Tissue was processed in 0.1% triton™ X-100 for protein extraction. Liquid chromatography-mass spectrometry (LC–MS) analysis of the tissue lysates was performed by nanoLC-MS/MS equipped with a Field Asymmetric Ion Mobility Spectrometry—FAIMS interface. This equipment is composed of a Vanquish Neo liquid chromatography system coupled to an Eclipse Tribrid Quadrupole, Orbitrap, Ion.

Each sample, containing 500 ng of peptides, was first introduced onto a trapping cartridge (Acclaim PepMap C18, 100 Å, 5 mm × 300 µm i.d., Thermo Scientific, Bremen, Germany) using a mobile phase composed of 2% acetonitrile (ACN) and 0.1% formic acid (FA) at a flow rate of 10 µL/min. After a 3-min loading phase, the trapped peptides were transferred to an analytical column (EASY-Spray, PepMap RSLC C18, 2 µm, 50 cm × 75 µm i.d., Thermo Scientific, Bremen, Germany) for separation at a flow rate of 250 nL/min. The separation was performed using a gradient elution, starting at 2.5% solvent B (80% ACN, 0.1% FA), increasing to 10% over 5 min, then to 30% over 120 min, 50% over the next 20 min, and finally reaching 99% over 5 min, which was maintained for an additional 10 min. The column was re-equilibrated at 2.5% B for 17 min. Data acquisition was conducted using Xcalibur 4.0 and Tune 2.9 software (Thermo Scientific, Bremen, Germany). 

The raw files were submitted to the Proteome Discoverer 3.0.1.27 software (Thermo Scientific, Bremen, Germany) for protein identification, using the Uniprot *Gallus gallus* Proteome database (43,711 entries, 2023_04). Total peptide amount was used to normalize abundance.

To conduct further functional analysis, differentially expressed protein between the experimental groups were identified, by applying the following filters: (a) a protein must be detected in at least 2 out 3 replicates of each experimental group; (b) at least two unique peptides per protein must be identified, with the *p* value adjusted using Benjamini–Hochberg correction for a false discovery rate (FDR) set to 0.01; (c) the HC-C/NG ratio was set to 1.5 for the selection of upregulated proteins and to 0.667 for downregulated proteins (Osório et al. 2021).

PANTHER 18.0 bioinformatics tools (https://www.pantherdb.org/) was used to perform protein classification. Proteins were classified according to gene ontology (GO) Biological Process annotations and REACTOME pathway annotations. FDR was calculated using the Benjamin-Hochberg method and set to be less than 0.05.

### Statistical analysis

Statistical analysis was performed using IBM® SPSS® Statistics (version 26.0, SPSS, USA). Mann–Whitney nonparametric test was applied, for group comparison to microtomographic analysis, ALP activity and carbonyl content assay results. To determine difference among groups in the Seahorse assay, an ANOVA with Tukey´s post-hoc test was conducted, after verifying data homoscedasticity with Levene´s test. Differences between groups were considered significant at *p* < 0.05.

## Results

### Bone tissue characterization

**Fig. 1:**
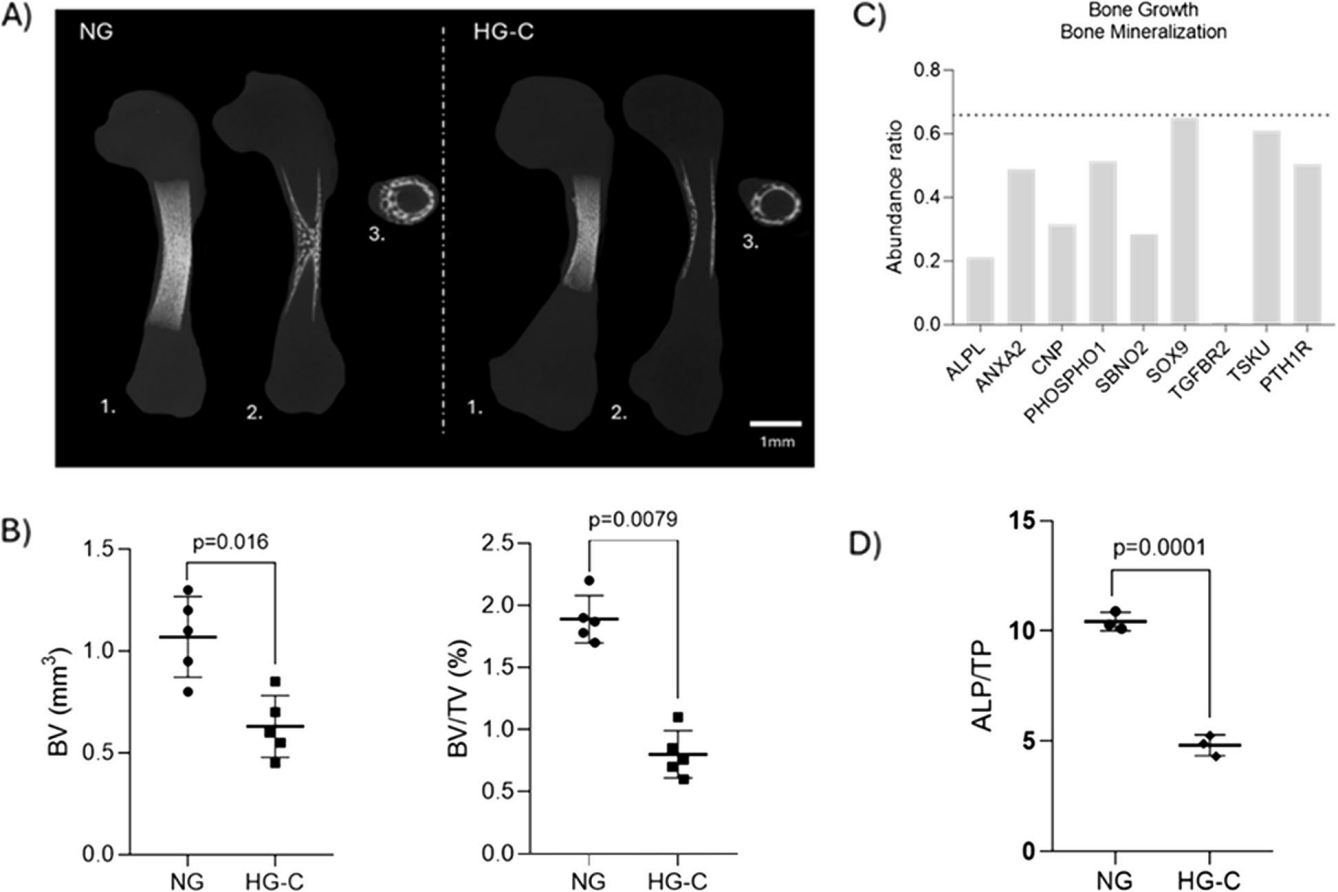
Bone tissue characterization. **A** Representative images of the microtomographic analysis of femora from NG and group HG-C – 1. Maximal projection of 3D reconstruction, 2. Axial cross section, and 3. Coronal cross section. Scale bar = 1 mm. **B** Histomorphometry analysis of microtomographic datasets. BV—bone volume, BV/TV – bone volume fraction. **C** Abundance ratio (HG-C/NG) represented on the Y axis for relevant proteins classified under Gene ontology-Biological process “Bone growth” and “Bone mineralization”, using PANTHER 18.0 bioinformatics tools. ALPL-alkaline phosphatase; ANX2-Annexin A2; CNP-C-type Natriuretic Peptide; CNP-C-type Natriuretic Peptide; PHOSPHO1-phosphatase orphan 1; SBNO2- Strawberry Notch Homolog 2; SBNO2- Strawberry Notch Homolog 2;SOX-9 -SRY-Box Transcription Factor 9; TGFBR2-Transforming Growth Factor Beta Receptor II ALPL-alkaline phosphatase; TSKU- Tsukushi; PHOSPHO1-phosphatase orphan 1; PTHR1-Parathyroid Hormone 1 Receptor. The dashed line at Y = 0.66 represents the threshold for significant expression differences, indicating downregulation of these proteins in the HG-C group. **D** Alkaline phosphate activity. The *p* values are indicated when the differences are found to be significant, *p* < 0.05

Microtomographic evaluation of NG and HG-C groups revealed a centripetal bone ingrowth at the mid-diaphysis, displaying a trabecular pattern evident in 3D reconstructions, as well as in axial and coronal cross sections (Fig. 1A). Compared to the NG group, the bone specimens maintained under HG-C conditions exhibited a marked reduction in mineralized content. Quantitative histomorphometric indices further supported these observations, evidencing a significant reduction in bone volume (BV) and bone volume fraction (BV/TV), in HG-C conditions (Fig. 1B).

The analysis of protein expression related to osteochondral development pathways highlighted a widespread decrease in relevant targets under HG-C conditions. Key regulators of chondrogenic differentiation and cartilage formation—crucial for endochondral ossification and skeletal development, such as SRY-Box Transcription Factor 9 (SOX9) and C-type Natriuretic Peptide (CNP), showed decreased levels. Additionally, there was a notable decrease in proteins involved in proliferation and differentiation, including Tsukushi (TSKU) and Strawberry Notch Homolog 2 (SBNO2). This reduction extended to essential phosphatases, such as alkaline phosphatase (ALPL) and phosphatase orphan 1 (PHOSPHO1). A decreased expression of Annexin A2 (ANXA2) was also verified. Furthermore, proteins involved in significant signaling pathways, including Transforming Growth Factor Beta Receptor II (TGFBR2) and Parathyroid Hormone 1 Receptor (PTH1R), exhibited decreased expression. All monitored proteins in the HG-C group fell below the threshold value of 0.66, signifying substantial downregulation in response to hyperglycemia (Fig. 1C). Moreover, alkaline phosphatase (ALP) activity of the sampled femora was significantly reduced in HG-C conditions, dropping to less than 50% of NG values (Fig. 1D).

Given that the mid-diaphysis region exhibited the most pronounced osteogenic activity and bone formation events, as evidenced within the microtomographic analysis, subsequent assays, including bioenergetic assessment and proteome characterization, were specifically focused on this region.

### Bone bioenergetics

The mitochondrial bioenergetics of embryonic chick mid-diaphysis femora were evaluated using a Seahorse Bioanalyzer, which measured the oxygen consumption rates (OCR) to assess mitochondrial function, and the extracellular acidification rate (ECAR) to evaluate glycolytic function. These analyses provided insights into both the basal and dynamic aspects of mitochondrial respiration, as well as glycolytic activity and capacity, through real-time assessments.

**Fig. 2:**
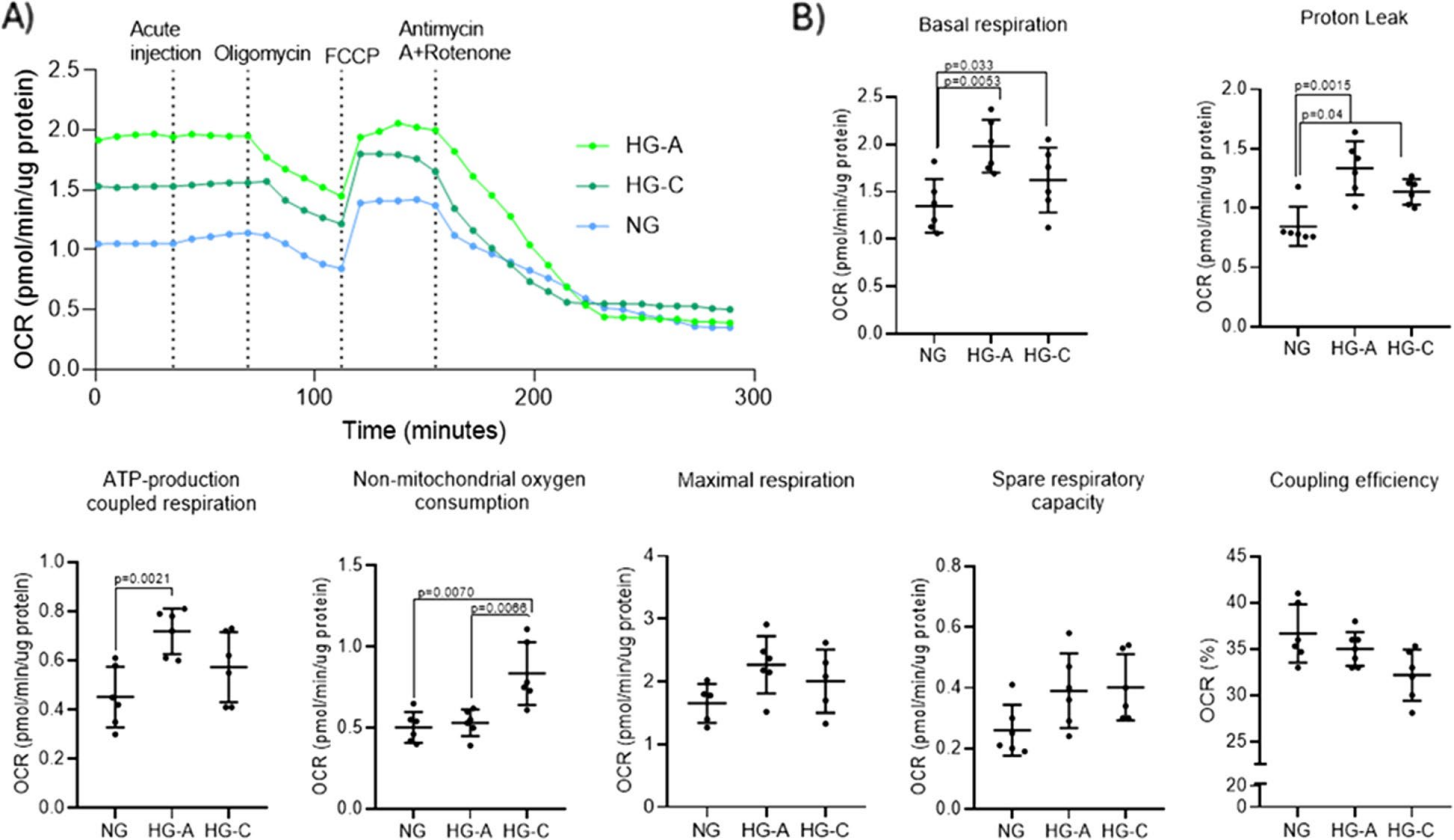
Bone bioenergetics – Mito Stress assay in NG, HG-A and HG-C groups. **A** Kinetic graph depicting real-time OCR. The compounds oligomycin (15 µM), FCCP (8 µM) and a mixture of rotenone (2 µM) and antimycin A (4 µM) were sequentially injected to modulate mitochondrial function. **B** Bioenergetic parameters derived from the Seahorse assay. Significant differences are indicated with *p* values (*p* < 0.05)

Following the evaluation of basal respiration and OCR under these conditions, a series of electron transfer chain (ETC) modulators were sequentially administered (Fig. 2A). Oligomycin, an inhibitor of complex V (ATP synthase), was used to assess ATP-linked OCR and calculate coupling efficiency, reflecting the efficiency of ATP production per oxygen molecule consumed. This was followed by the addition of FCCP (carbonyl cyanide-4-(trifluoromethoxy) phenylhydrazone), an uncoupler used to determine the maximal respiratory capacity, providing insight into the mitochondria's potential under stress. The difference between the maximal respiratory capacity and basal respiration, known as the spare respiratory capacity, serves as an indicator of the cell’s capacity to respond to increased metabolic demands. Proton leak measurements were also taken to indicate the non-ATP-producing oxygen consumption. To assess the contribution of non-mitochondrial sources to cellular respiration, a combination of rotenone (complex I inhibitor) and antimycin A (complex III inhibitor) was administered, effectively blocking electron transport through the ECT and isolating the OCR derived from non-mitochondrial enzymatic processes (Bessa et al. 2023).

Comparatively to NG conditions, both hyperglycemic states (HG-A and HG-C) elicited a significantly higher basal respiration rate, with HG-A demonstrating elevated levels relative to HG-C. This trend was similarly observed for proton leak measurements, in which HG-A reached the highest values, followed by HG-C, with both significantly exceeding those recorded under NG conditions. Regarding ATP-production coupled respiration, HG-A showed significantly higher levels, whereas HG-C did not differ significantly from NG. Conversely, non-mitochondrial oxygen consumption in HG-C increased significantly in relation to both NG and HG-A. No significant differences were observed in the remaining parameters, including spare respiratory capacity, maximal respiration and coupling efficiency (Fig. 2B).

**Fig. 3:**
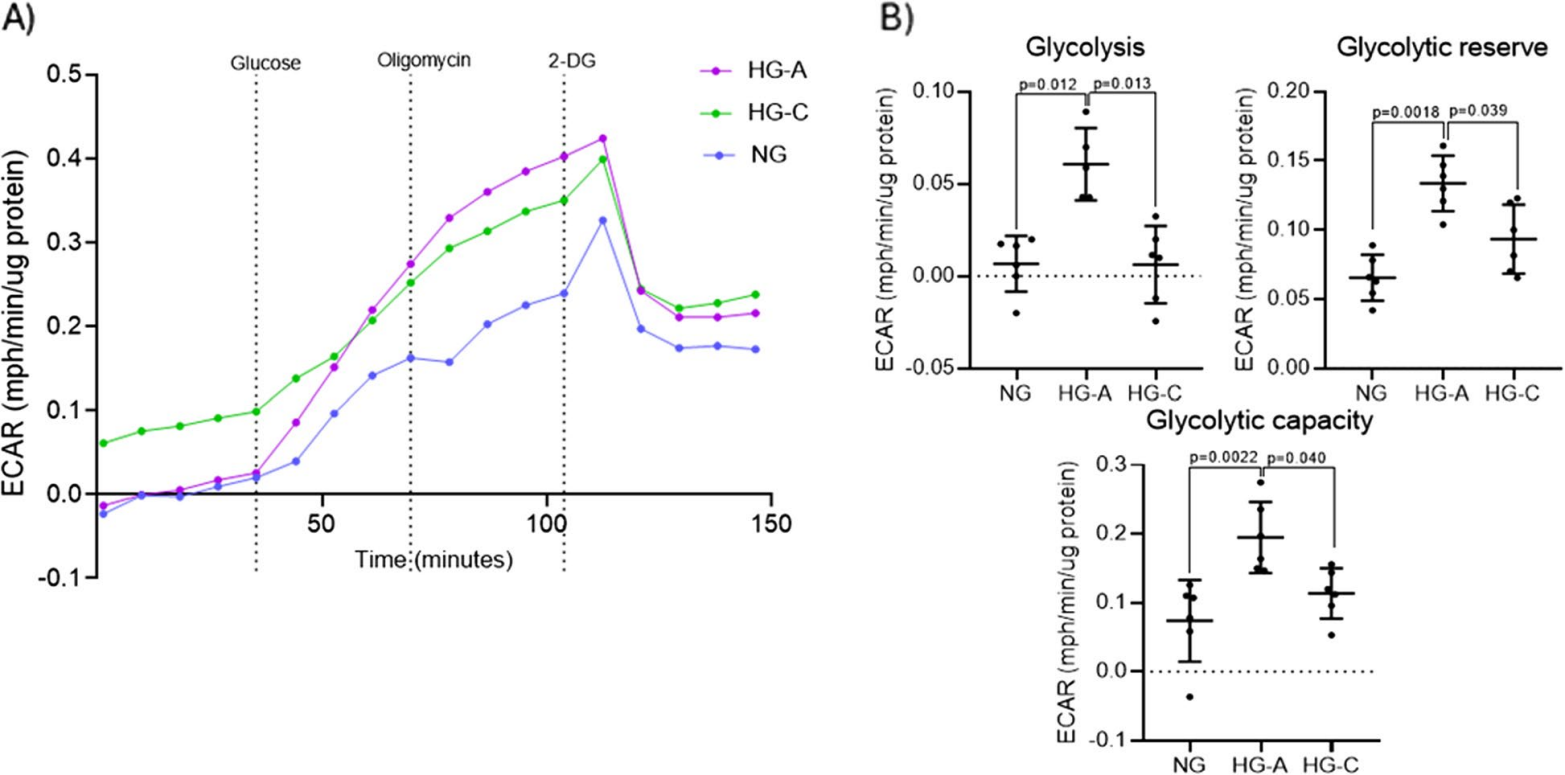
Bone bioenergetics – Glycolysis Stress Test in NG, HG-A and HG-C groups. **A** Kinetic graph depicting real-time ECAR. The compounds oligomycin (15 µM) and 2DG (100 mM) were sequentially injected to assess glycolytic function (**B**) Bioenergetic parameters derived from the Seahorse assay. Significant differences are indicated with *p* values (*p* < 0.05)

Glycolysis stress test measures the extracellular acidification rate (ECAR) caused by the glycolytic process (Fig. 3A), upon glucose exposure, ascertaining the cells’ capacity to perform glycolysis. In addition, the ability to resource to glycolysis – glycolytic capacity- when mitochondrial energetic is compromised, is evaluated upon exposure to oligomycin. The difference between the glycolytic capacity and basal glycolysis is denominated glycolytic reserve, indicating the cells´ ability to respond to high energetic demand by increasing glycolysis. The exposure to 2-DG, that blocks the glycolytic process, elucidates on the acidification rate that is not linked to glycolytic breakdown (Bessa et al. 2023).

The HG-A group exhibits a significant increase in glycolysis, glycolytic capacity and glycolytic reserve compared to NG group. Conversely, the HG-C group showed a reversal of this trend, with significant reductions in all parameters compared to HG-A group, but not showing significant differences compared to the NG group (Fig. 3B).

### Proteome assessment

**Fig. 4:**
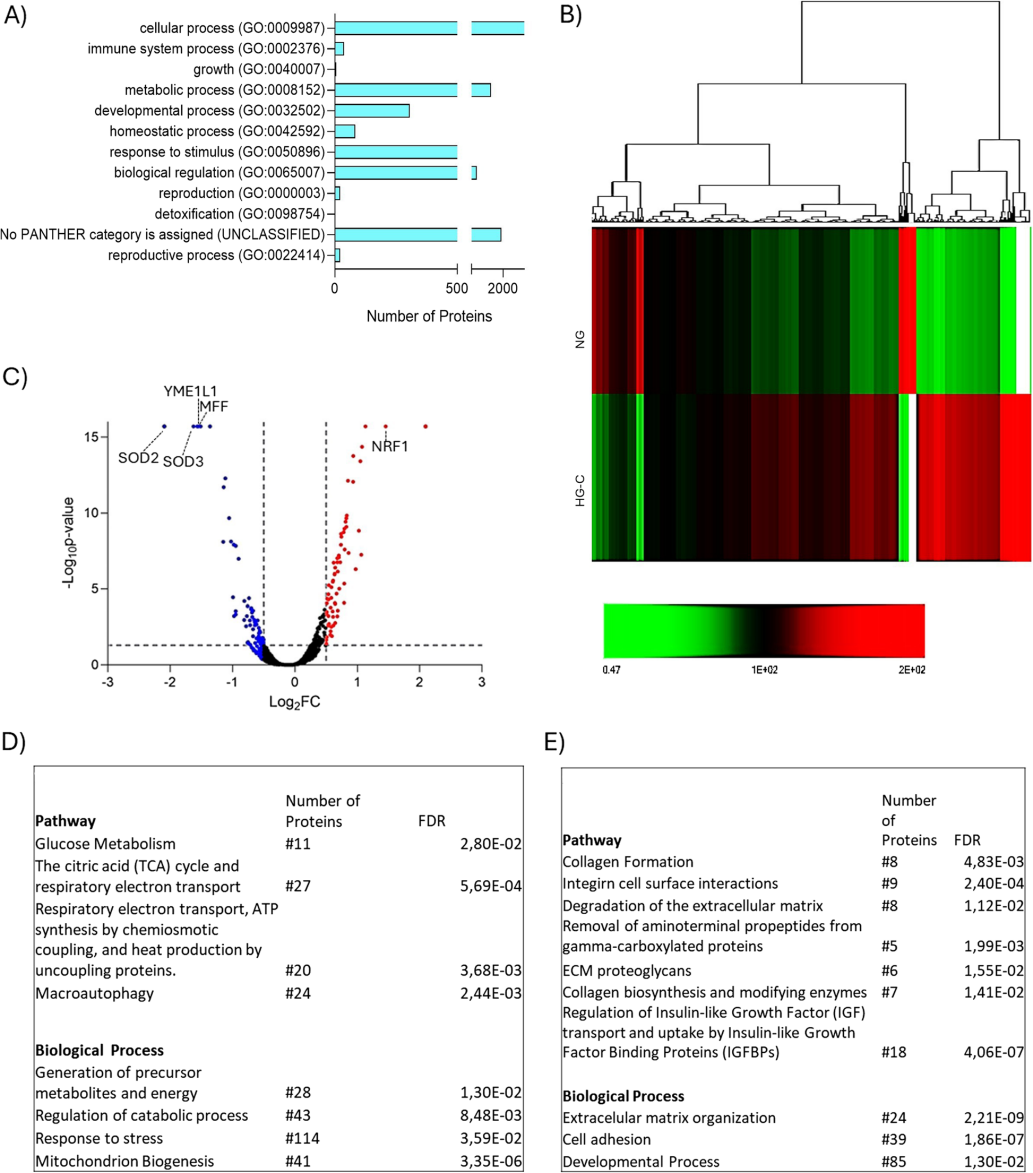
Proteome characterization. **A** Protein classification overview of the confidently identified proteins through LC/MS (FDR < 0.01), categorized according to Gene ontology—Biological Process using PANTHER 18.0 bioinformatics resources. **B** Heat map illustrating the quantitative distribution of protein relative abundance (averaged data, *n* = 3) between NG and HG-C groups. **C** Volcano plot showing the distribution of differentially expressed proteins in HG-C group. Blue dots represent downregulated proteins in the HG-C group (FC < 0.6, FDR < 0.05), while red dots represent upregulated proteins in HG-C group (FC < 1.5, FDR < 0.05). **D** Table showing the classification of upregulated proteins in HG-C group, according to Reactome pathways and biological processes, using PANTHER 18.0 bioinformatics tools. **E** Table showing the classification of downregulated proteins in HG-C group, according to Reactome pathways and biological processes, using PANTHER 18.0 bioinformatics tools

The proteome characterization was performed using liquid chromatography-mass spectrometry (LC/MS), confidently identifying 5,876 proteins. Using PANTHER 18.0 bioinformatic tools, a comprehensive protein classification was conducted, providing an overview of the functionality of the proteome (Fig. 4A). This analysis matched 3,918 proteins to Gene Ontology—Biological Process terms, predominantly in cellular process (2,874 proteins), biological regulation (1,371 proteins), and metabolic process (1,711 proteins). However, PANTHER was unable to classify 1,958 proteins (Fig. 4A).

Despite the high similarity in overall proteome classification between NG and HG-C groups, the heat map (Fig. 4B) illustrates a distinct protein expression profile between the two groups. Identification of differentially expressed proteins was performed, and their distribution is shown in the volcano plot (Fig. 4C). This analysis revealed approximately 226 proteins that were significantly downregulated, and 204 proteins that were significantly upregulated in the HG-C group. Further analysis of these differentially expressed proteins through pathway enrichment and biological process classification underscored their functional implications (Fig. 4D and E). Notably, significantly downregulated proteins in the HG-C group are mostly related to cellular metabolism, particularly energetic processes (Fig. 4E). These include proteins associated with the following Reactome pathways: glucose metabolism (11 proteins); the citric acid (TCA) and respiratory electron chain (27 proteins); respiratory electron transport, ATP synthesis by chemiosmotic coupling, and heat production by uncoupling proteins (20 proteins); and macroautophagy (24 proteins). Biological process classification identified 114 significantly downregulated proteins in the HG-C group associated with response to stress, along with 41 proteins involved in mitochondrion biogenesis. Conversely, overexpressed pathways in the HG-C group (Fig. 4D) broadly relate to collagen synthesis and maturation, and extracellular matrix organization.

**Fig. 5:**
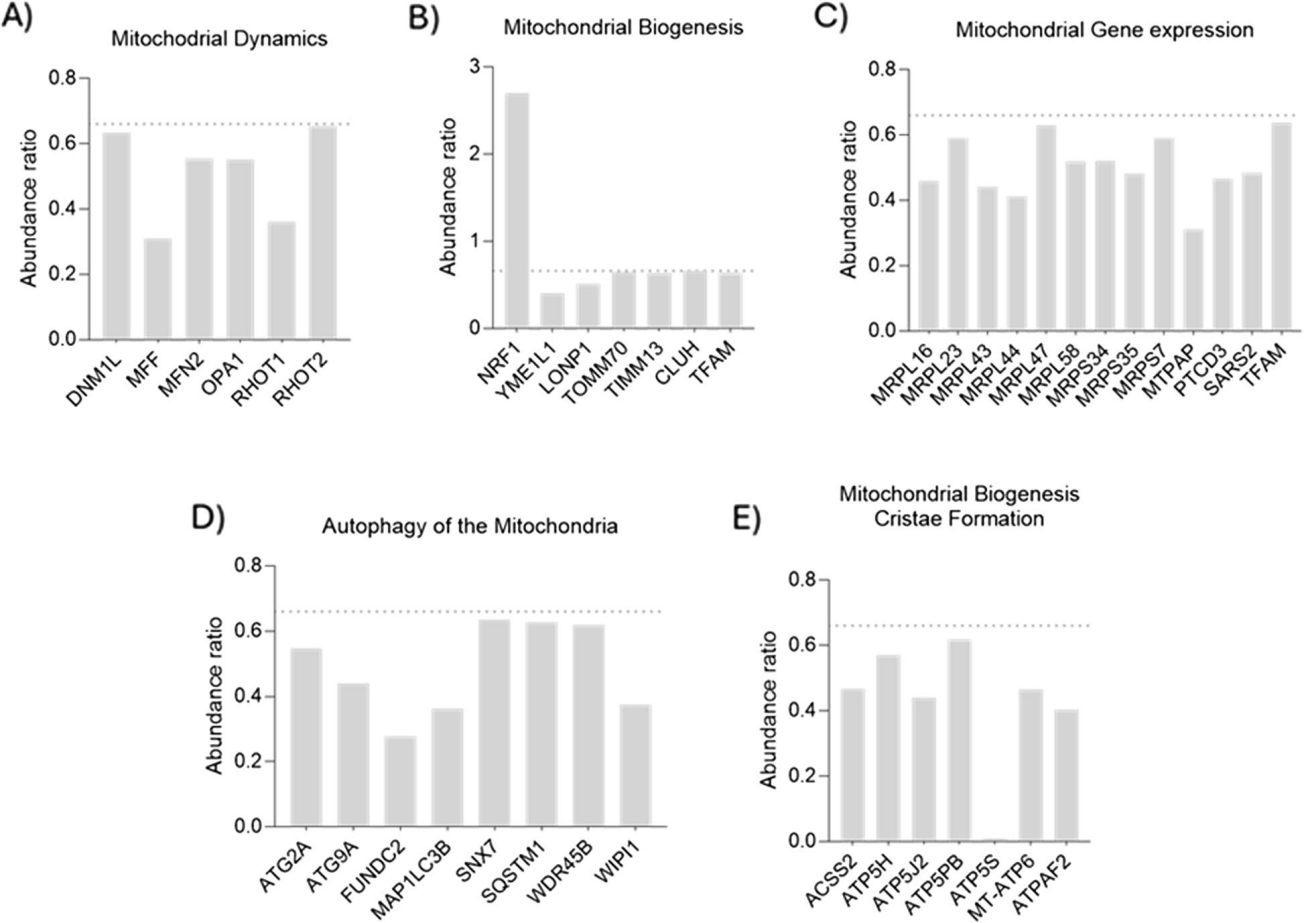
Assessment of protein expression relevant for mitochondrial function. Abundance ratio HG-C/NG is considered significant if it is over 1.5 or less than 0.66, with an adjusted *p* value < 0.05. Proteins were classified under Gene ontology – Biological process or Reactome pathway, using PANTHER 18.0 bioinformatics tools. Figure highlights differentially expressed proteins critical for mitochondrial dynamics, biogenesis, gene expression, autophagy/mitophagy, and ATP synthesis. MNF2-mitofusin-2; DNM1L-dynamin-1-like; MFF- mitochondrial fission factor; RHOT1-mitochondrial Rho GTPase 1; OPA 1- mitochondrial dynamin like GTPase; RHOT2- mitochondrial Rho GTPase 2; NRF1- nuclear respiratory factor 1; YME1L1-YME1 like 1 like ATPase; LONP1-lon peptidase 1; TOMM70- translocase of outer mitochondrial membrane 70, TIMM13-translocase of inner mitochondrial membrane 13; CLUH-clustered mitochondrial homolog; TFAM-mitochondrial transcription factor A; MRPL16, MRPL23, MRPL43, MRPL44, MRPL47, MRPL58—mitochondrial ribosomal large subunit proteins; MRPS34, MRPS35, MRPS7- mitochondrial ribosomal small subunit proteins; MTPAP-mitochondrial poly(A) polymerase; PTCD3- mall ribosomal subunit protein mS39; SARS2- mitochondrial Seryl-tRNA synthetase 2;TFAM-mitochondrial transcription factor A; ATG2 A-autophagy-related 2 A; ATG9- autophagy-related protein 9; FUNDC2-FUN14 domain containing 2; MAP1LC3B-microtubule-associated proteins 1 A/1B light chain 3B; SNX7- sortin nexin 7; SQSTM1-sequestosome 1; WDR45B- WD repeat domain phosphoinositide-interacting protein 3; WIPI1-WD repeat domain phosphoinositide-interacting protein 1; ACSS2- acyl-CoA synthetase short chain family member 2; ATP5H-ATP synthase, H + transporting, mitochondrial Fo complex subunit D; ATP5 J2- ATP synthase, H + transporting, mitochondrial Fo complex subunit F2; ATP5B ATP synthase peripheral stalk-membrane subunit b; ATP5S-ATP synthase, H + transporting, mitochondrial Fo complex subunit s ATP synthase, H + transporting, mitochondrial Fo complex subunit s; MT-APT6- mitochondrially encoded ATP synthase 6; ATPAF2-ATP synthase mitochondrial F1 complex assembly factor 2

A distinct expression pattern of proteins related to mitochondrial functionality was observed in the HG-C group. Proteins involved in mitochondrial dynamics were significantly reduced under hyperglycemic conditions (Fig. 5A). Notably, the levels of OPA 1 mitochondrial dynamin like GTPase (OPA1) and mitofusin-2 (MFN2), which are crucial for mitochondrial fusion, were markedly decreased, suggesting impaired fusion processes. Additionally, a significant reduction in dynamin-1-like protein (DNM1L) and mitochondrial fission factor (MFF) levels suggests compromised mitochondrial fission. Furthermore, the decreased levels of mitochondrial Rho GTPase 1 (RHOT1) and mitochondrial Rho GTPase 2 (RHOT2) indicate diminished mitochondrial transport and mobility activities, collectively highlight a disruption in the balance of mitochondrial dynamics.

In the context of mitochondrial biogenesis (Fig. 5B), and while nuclear respiratory factor 1 (NRF1), which plays a crucial role in regulating targets in mitochondrial DNA transcription and replication, was highly expressed under hyperglycemic conditions, several key proteins associated with mitochondrial biogenesis and maintenance were significantly reduced. These proteins include clustered mitochondrial homolog (CLUH), translocase of outer mitochondrial membrane 70 (TOMM70), translocase of inner mitochondrial membrane 13 (TIMM13), lon peptidase 1 (LONP1), and YME1 like 1 like ATPase (YME1L1) (Fig. 5B). Additionally, transcription factor A (TFAM) and LONP1, involved in the regulation and maintenance of mitochondrial DNA, also showed reduced levels (Fig. 5C). Mitochondrial ribosomal proteins, crucial for synthesizing proteins encoded by mitochondrial DNA, were also affected, further contributing to an acknowledged impaired mitochondrial function.

Mitophagic and autophagic processes (Fig. 5D) were also acknowledged to be impaired. Notably, sequestosome 1 (SQSTM1) and microtubule-associated proteins 1 A/1B light chain 3B (MAP1LC3B), both essential for autophagosome formation, were significantly reduced. Additionally, autophagy-related 2 A (ATG2 A) and WD repeat domain phosphoinositide-interacting protein 1 (WIPI1), which are involved in the initiation of autophagy, were downregulated. FUN14 domain containing 2 (FUNDC2), a protein that mediates mitophagy under hypoxic conditions, also showed reduced levels, supporting an impaired mitophagy process.

In Complex V (ATP synthase), there was a significant reduction in the expression of several ATP synthase subunits (Fig. 5E), including acyl-CoA synthetase short chain family member 2 (ACSS2), ATP synthase, H + transporting, mitochondrial Fo complex subunit D (ATP5H), ATP synthase, H + transporting, mitochondrial Fo complex subunit F2 (ATP5 J2), ATP synthase peripheral stalk-membrane subunit b (ATP5PB), ATP synthase, H + transporting, mitochondrial Fo complex subunit s ATP synthase, H + transporting, mitochondrial Fo complex subunit s (ATP5S), and mitochondrially encoded ATP synthase 6 (MT-ATP6), along with ATP synthase mitochondrial F1 complex assembly factor 2 (ATPAF2).

### Oxidative stress assessment

**Fig. 6:**
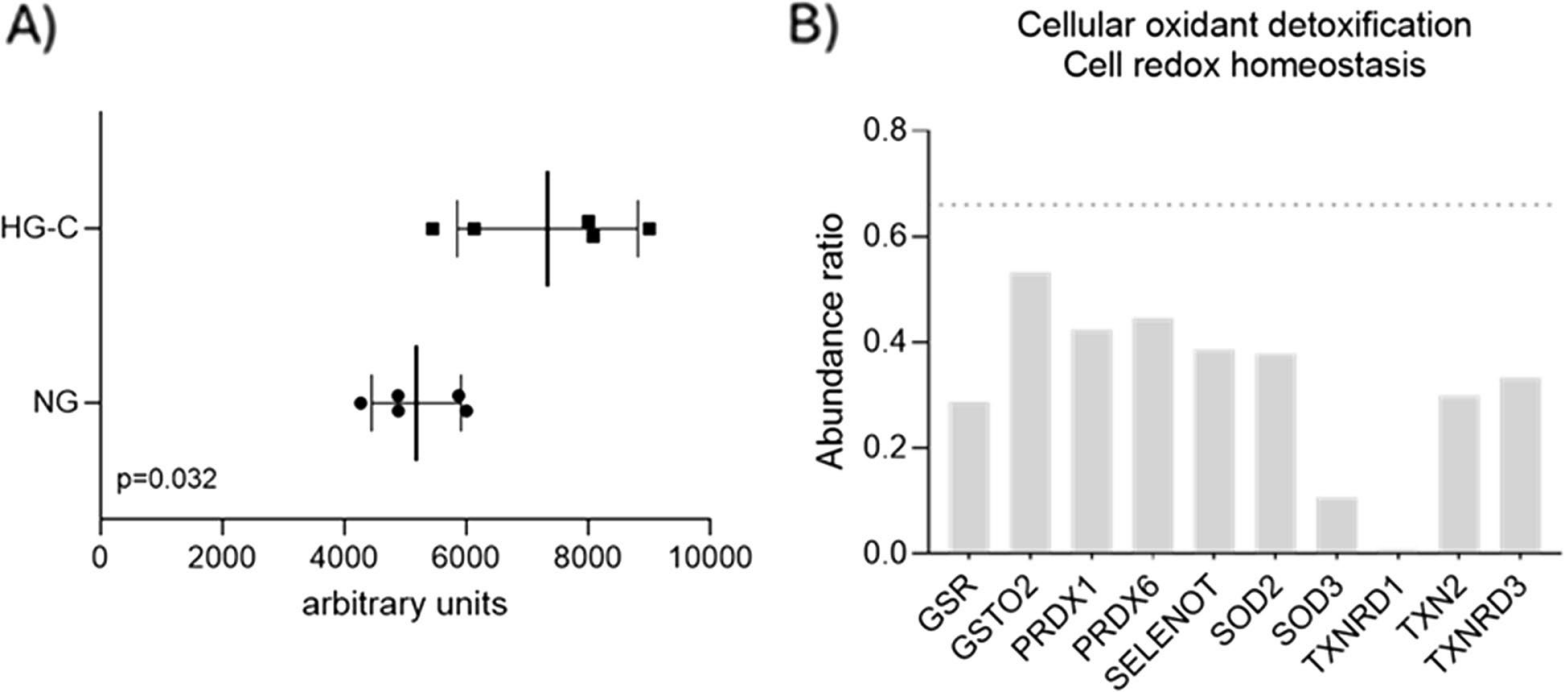
Carbonyl content assay results, differences between groups were found to be significant. B) Protein expression of targeted proteins associated with the cellular oxidant detoxification. Abundance ratio HG-C/NG is considered to be significant if over 1.5 or less than 0.66, with adjusted p-value<0.05. Proteins were classified under Gene ontolology –Biological process or Reactome pathway, using PANTHER 18.0 bioinformatics tools. GSR-glutathione reductase; GSTO2-glutathione S-transferase Omega 2; PRDX1-peroxiredoxin 1; PRDX6- peroxiredoxin 6; SELENOT- thioredoxin reductase-like selenoprotein T; SOD2- superoxide dismutase2; SOD3- superoxide dismutase 3; TXRND1-thioredoxin reductase 1; TXN2-thioredoxin; TXNRD3-thioredoxin reductase 3

The determination of carbonyl content showed significantly higher levels in the HG-C group compared to the NG group (Fig. 6A). Carbonyls, produced as a consequence of structural protein damage via oxidative stress, underscore the cumulative impact of persistent reactive oxygen species (ROS) generation following glucose exposure. Furthermore, a pronounced reduction in the expression of proteins pivotal to the cellular oxidant detoxification and redox homeostasis was verified within the HG-C group (Fig. 6B). Within the glutathione system, both glutathione reductase (GSR) and glutathione S-transferase Omega 2 (GSTO2) showed reduced expression, indicating compromised glutathione recycling and detoxification capacity. The peroxiredoxin family, including PRDX1 and PRDX6, also displayed decreased abundance, suggesting impaired peroxide detoxification. Selenoproteins, such as thioredoxin reductase-like selenoprotein T** (**SELENOT), exhibited reduced levels, further impacting antioxidant defense mechanisms. The superoxide dismutase (SOD) family, including SOD2 and SOD3, was significantly downregulated, leading to decreased dismutation of superoxide radicals. Additionally, components of the thioredoxin system, such as thioredoxin reductase 1 (TXNRD1), thioredoxin (TXN), and thioredoxin reductase 3 (TXNRD3), showed reduced expression, highlighting a broad impairment in maintaining cellular redox balance.

## Discussion

Diabetic bone disease is characterized by significant metabolic and structural alterations within bone tissue, leading to an elevated fracture risk and impairing healing capabilities (Balint et al. 2001). Despite the extensive use of diverse models, from clinical datasets to experimental in vitro and in vivo approaches, the precise mechanistic drivers underlying these alterations remain largely unknown. Within this metabolic framework, a critical yet conspicuously absent aspect in current literature is comprehending how hyperglycemia perturbs bioenergetic processes in bone and affects metabolic functionality. This study aims to address this knowledge gap.

Using an established translational ex vivo model of bone in hyperglycemic conditions with embryonic chick femurs exposed to high glucose conditions, the study observed that chronic exposure led to decreased bone formation and reduced mineral content, as evidenced through microtomographic assessment. This strongly indicates impaired osteogenesis, likely due to disrupted cellular differentiation and maturation processes (Araújo et al. 2022; Komori 2006). Proteomic analysis revealed the downregulation of key proteins categorized under “Bone growth/mineralization” in the Gene Ontology-Biological Process classification. Proteins associated with proliferation and differentiation events, such as TSKU (Yano et al. 2017) and SBNO (Syme et al. 2022) were downregulated, indicating a broad impairment in the cellular mechanisms essential for bone growth. Additionally, downregulated proteins crucial for chondrogenic differentiation and cartilage formation, such as SOX9 and CNP, suggested a hindrance in endochondral ossification (Galea et al. 2021). A significant decrease in the expression of phosphatases, including ALPL and PHOSPHO1, essential for providing phosphate for hydroxyapatite formation (Huesa et al. 2015) implied an impediment to the mineralization process, compounded by reduced expression of mineral nucleation proteins like Annexin 2 (Klabklai et al. 2022). Moreover, diminished levels of key proteins in the TGF-β and PTH signaling pathways, evidenced by reduced levels of TGFBR2 and PTH1R, suggest these pathways may contribute to the observed effects, as previously demonstrated (Roy 2013). These findings align with known molecular alterations induced by hyperglycemia and are consistent with reported changes in diabetic bone tissue (Wongdee and Charoenphandhu 2011), underscoring the effectiveness of this ex vivo model in elucidating comprehensive and translatable alterations associated with diabetic bone disease (Araújo et al. 2022).

The bone tissue bioenergetics were comprehensively assessed using a Seahorse Extracellular Flux Analyzer, incorporating both the mitochondrial stress test and glycolysis stress test. To the best of the authors’ knowledge, no prior studies have explored the bioenergetic profile of bone tissue under either normoglycemic or hyperglycemic conditions; previous investigations have been limited to differentiating osteoblastic populations (Medeiros and Wallace 2022). Under physiological glucose levels, these studies revealed a unique reliance on glycolysis for ATP production, even in the presence of oxygen—a phenomenon known as the Warburg effect (Esen and Long 2014). This metabolic adaptation enables differentiating osteoblasts to rapidly generate ATP and essential metabolic intermediates required for bone matrix production and mineralization (Esen and Long 2014). Despite yielding less ATP than oxidative phosphorylation, glycolysis provides a quicker energy source, supporting the substantial energetic demands associated with active bone formation (Esen and Long 2014). This distinctive metabolic profile highlights the unique bioenergetics of the bone tissue compared to other cell types and tissues, which primarily rely on oxidative phosphorylation for energy production (Riddle and Clemens 2017).

To investigate the effects of high glucose conditions on the bioenergetic profile of bone tissue, femurs were subjected to both chronic (HG-C) and acute (HG-A) high glucose exposure. In both HG conditions, there was a notable increase in basal respiration rate and proton leak, indicating shifts in the mitochondrial bioenergetic profile due to elevated glucose levels. This contrasts with observations from various other tissues and cells exposed to high glucose levels in vitro, typically within the 20 mM to 30 mM range, where chronic exposure (e.g., in podocytes, endothelial cells, myotubes, epithelial cells, pancreatic cells, and aortic tissues) often resulted in basal respiration rates that either remained unchanged or were reduced (Audzeyenka et al. 2021; Chen et al. 2019; Lund et al. 2019; Haythorne et al. 2019). However, these results align with previous findings from osteoblastic cell cultures exposed to acute high glucose levels (Medeiros and Wallace 2022), reinforcing the notion that these differences may be attributed to the unique bioenergetic profile of bone tissue (Esen and Long 2014).

Further analysis revealed that both HG-A and HG-C conditions led to a significant increase in basal respiration, sustaining the electrochemical potential within the ETC. Notably, after acute exposure (HG-A) the increased oxygen consumption appears to be driven by ATP production, as evidenced by the increased ATP-production coupled respiration (Bessa et al. 2023), further supporting that additional glucose availability drives mitochondrial function. In contrast, chronic exposure (HG-C) oxygen consumption did not fully align with tissue metabolic demands, as indicated by the fact that this was driven by the increased non-mitochondrial oxygen consumption instead of ATP-production coupled respiration. This suggests that chronic high glucose exposure may compromise mitochondrial energy production efficiency, potentially reflecting the instalment of mitochondrial dysfunction as end result or as an adaptation to cope with the need to mitigate prolonged oxidative stress (Nishikawa et al. 2000).

The increase in proton leak observed under both HG-A and HG-C conditions implies a heightened state of mitochondrial uncoupling in response to high glucose. Proton leak, the process by which protons cross the mitochondrial membrane without contributing to ATP synthesis, can be an adaptive response to mitigate the detrimental effects of excessive ROS production (Nishikawa et al. 2000). This uncoupling can help reduce oxidative damage by preventing the over-reduction of the electron transport chain and subsequent ROS generation. However, it comes at the cost of reduced efficiency in ATP production. In the HG-A group, despite the increase in proton leak, the mitochondria were still able to maintain high ATP production, suggesting that acute glucose levels primarily induce a compensatory mechanism to manage immediate oxidative stress without severely compromising energy output (Bessa et al. 2023). On the other hand, in the HG-C group, the increased proton leak did not translate into higher ATP production, indicating that chronic hyperglycemia may lead to more substantial mitochondrial dysfunction (Cheng et al. 2022).

The glycolysis stress assay demonstrated a distinct response in both HG-A and HG-C conditions. HG-A exposure led to increased glycolysis and glycolytic capacity, resulting in an increased glycolytic reserve (Bessa et al. 2023). This suggests an upregulation of the glycolytic pathway as a rapid adaptation to glucose surplus. Overall, HG-A exposure induced both glycolysis and OXPHOS, ensuring a swift and efficient energy production response by leveraging the Warburg effect—characteristic of bone tissue (Esen and Long 2014). Importantly, this metabolic adaptation did not correlate with a diminution of mitochondrial function, indicating that acute exposure to high glucose triggers a robust and synergistic activation of both glycolytic and oxidative pathways (Esen and Long 2014). In contrast, chronic exposure (HG-C) primarily increased non-mitochondrial oxygen consumption without significant changes in glycolytic pathway parameters. This indicates that, although the tissue initially adapts to acute glucose surplus by upregulating both energy production pathways, prolonged high glucose exposure appears to uncouple the oxygen consumption from energy production. This shift may result from the saturation or downregulation of glycolytic pathways, reflecting an adaptive response to prolonged oxidative stress and sustained strain of the metabolic pathways (Mussi et al. 2022). Additionally, the persistent elevation in non-mitochondrial oxygen consumption and proton leak under HG-C conditions may indicate increased ROS production and oxidative damage, further challenging the bioenergetic efficiency of bone cells (Mussi et al. 2022).

To explore the proteomic changes induced by HG exposure, liquid chromatography-mass spectrometry (LC/MS) and bioinformatic analysis were conducted on bone samples, revealing shifts in protein expression profiles. Although the overall proteome classification between physiological glucose levels (NG) and HG-C groups was similar, distinct expression patterns emerged. Notably, the HG-C group exhibited a significant downregulation of proteins associated with cellular metabolism, while pathways related to collagen synthesis and extracellular matrix organization were upregulated. Given these findings and the altered bioenergetic profile indicative of mitochondrial dysfunction, a detailed investigation into mitochondrial functionality was undertaken.

Upon HG exposure, a decreased expression of key proteins associated with mitochondrial biogenesis (CLUH, TOMM70, TIMM13, LONP1, and YME1L1) and those associated with the regulation of mitochondrial DNA (TFAM and LONP1) were verified. Despite this overall decrease, an increased expression of NRF-1, a nuclear transcription factor and downstream target of the master regulator of mitochondrial biogenesis—PGC-1α, was identified. NRF-1 is essential for enhancing the transcription of proteins and enzymes involved in various mitochondrial activities, including mitochondrial biogenesis, maintenance of mitochondrial DNA, oxidative phosphorylation, and the regulation of respiratory chain complexes (Choi et al. 2004). Typically, NRF-1 expression is reduced in diabetic and high glucose conditions, with its increase often associated with therapy-induced metabolic recovery (Ding et al. 2024). Nonetheless, elevated NRF-1 under high glucose has been documented, such as in the brain of hyperglycemic animals (Kumari et al. 2012) and in muscle cells exposed to high glucose (Choi et al. 2004; Kumari et al. 2012). While elevated NRF-1 expression in HG conditions enhanced glucose transport by upregulating transporters like GLUT4, this does not necessarily translate into improved mitochondrial functionality (Baar et al. 2003). Consistently with the present data, the increase in NRF-1 may be a compensatory response to high glucose stress aimed at mitigating mitochondrial dysfunction. However, without a coordinated upregulation of other mitochondrial biogenesis proteins, this isolated increase in NRF-1 is insufficient to fully restore mitochondrial function and efficiency (Baar et al. 2003).

In addition to the altered biogenesis under HG-C exposure, evidence of impaired mitochondrial dynamics was observed. There was a downregulation of key fusion proteins (MFN2 and OPA1) (Westermann 2010) and fission proteins (MFF and DNM1L) (Westermann 2010; Westermann 2012). Furthermore, reduced mitochondrial mobility was indicated by the downregulation of RHOT1 and RHOT2 (Zaninello and Bean 2023), while proteins related to autophagy (SQSTM1, MAP1LC3B, ATG2 A) and mitophagy (FUNDC2) (Fan et al. 2023) were also under-expressed. Mitochondria undergo continuous fusion and fission processes to maintain the organelle morphology and functionality, remove damaged mitochondria, and regulate cell death pathways (Youle and Bliek 2012). These dynamic processes ensure proper mitochondrial distribution, optimize energy production, and facilitate cellular responses to metabolic demands and stress (Chen et al. 2023). Hyperglycemic conditions have been associated with disturbed mitochondrial dynamics, as evidenced by impaired fission/fusion processes in the cells of different tissues (Alka et al. 2023; Rovira-Llopis et al. 2017), affecting cellular quality control and bioenergetic capability (Liesa and Shirihai 2013). Increased mitochondrial immobilization due to reduced mobility has also been identified upon high glucose exposure, given the modulation of the mitochondrial motor-adaptor complex and enhancement of mitochondrial anchoring to the actin cytoskeleton (Zaninello and Bean 2023). Consistent with the reports of hindered mitochondrial dynamics, mitophagy – the process of clearing damaged mitochondria through autophagy – was found to be inhibited under chronic HG conditions via the AMPk/mTOR signaling (Wang et al. 2020). This inhibition is expected to result in the accumulation of damaged mitochondria, leading to increased cellular stress and reduced metabolic efficiency (Zheng et al. 2021). Overall, the observed disrupted mitochondrial lifecycle in the bone tissue exposed to HG is in line with observations reported in various other tissues exposed to HG. This disruption is known to contribute to cellular injury and potential excessive ROS generation (Zheng et al. 2021).

Regarding the mitochondrial functionality, the most relevant observation refers to the reduced expression of mitochondrial complex V ATP synthase subunits – ATP5H, ATP5B, ATP5, ATP5 J2 under chronic high glucose exposure, which is suggestive of an impaired ATP production in response to microenvironmental changes, as evidenced by the seahorse data. ATP-synthase drives the phosphorylation of ADP into ATP, due a conformational shift that is mediated by the electrochemical gradient generated by the electron transfer through mitochondrial complexes I to IV (Nishikawa et al. 2000). This observation supports that despite increased oxygen consumption that drives OXPHOS under chronic hyperglycemia, it does not translate into superior energetic balance, as the final step in ATP production might be compromised. Furthermore, evidence of an increased level of oxidative stress in bone tissue exposed to HG conditions was indicated by significantly higher carbonyl content – a stable marker of oxidative protein modification – and reduced expression of proteins associated with detoxification and redox homeostasis, such as those in the glutathione and thioredoxin systems, peroxiredoxins, and superoxide dismutases (Akagawa 2021). The increased proton leak observed under HG-C conditions, indicating heightened oxygen consumption uncoupled from ATP production, can contribute to elevated ROS production through several mechanisms. These include enhanced activity at complexes I and III of the ETC, involvement of antiporter proteins, and activation of uncoupling proteins (Mailloux 2018). In addition to mitochondria-mediated ROS production, the expected activation of alternative metabolic pathways – such as the polyol and hexosamine pathways – due to excess glucose (Mailloux 2018), correlates with the observed increase in non-mitochondrial oxygen consumption in the HG-C groups. This activation can further contribute to the heightened ROS production, alongside glucose auto-oxidation and the formation of advanced glycation end products (AGEs) (Mailloux 2018). The increased ROS accumulation is exacerbated by the diminished activity of detoxification proteins of the different systems, impairing the cell’s ability to neutralize ROS and leading to further oxidative damage and cellular dysfunction.

Although high-glucose exposure in this study was limited to a single concentration, the findings contribute to a deeper understanding of how bioenergetic alterations may influence bone tissue dysfunction. While this organotypic model does not fully replicate the systemic complexity of diabetes mellitus, it provides valuable insights into the direct effects of prolonged hyperglycemia on bone metabolism. The observed bioenergetic disruptions align with mechanisms implicated in diabetic bone disease, supporting the relevance of this model in studying tissue-level responses that may also occur in vivo (Araújo et al. 2022).

## Conclusion

This study demonstrated that chronic hyperglycemia leads to significant bioenergetic and proteomic alterations in bone tissue. Bioenergetic assessment revealed increased basal respiration rates and proton leak under both acute and chronic exposure to high glucose. However, chronic exposure led to increased non-mitochondrial oxygen consumption and unchanged ATP production, in result of compromised mitochondrial efficiency and impaired glycolytic function. In contrast, acute high glucose exposure maintained higher ATP production despite increased proton leak, suggesting a compensatory mechanism to manage immediate oxidative stress. Proteomic analysis further supported these findings, showing downregulation of key proteins involved in mitochondrial biogenesis, dynamics, and detoxification. Increased oxidative stress was indicated by higher carbonyl content and reduced expression of proteins critical for maintaining redox homeostasis.

These results highlight the extensive metabolic and bioenergetic disruptions induced by chronic high glucose exposure, emphasizing a continuous across spectrum of deleterious effects on mitochondrial function, in bone tissue. This underscores the need for targeted strategies aimed at reducing oxidative stress and restoring mitochondrial function in order to prevent diabetic bone disorder or improve bone health in affected individuals.

## Data Availability

The data that support the findings of this study are not openly available due to reasons of sensitivity and are available from the corresponding author upon reasonable request.
